# The Role of Anterior Nuclei of the Thalamus: A Subcortical Gate in Memory Processing: An Intracerebral Recording Study

**DOI:** 10.1371/journal.pone.0140778

**Published:** 2015-11-03

**Authors:** Klára Štillová, Pavel Jurák, Jan Chládek, Jan Chrastina, Josef Halámek, Martina Bočková, Sabina Goldemundová, Ivo Říha, Ivan Rektor

**Affiliations:** 1 Central European Institute of Technology CEITEC, Brain and Mind Research Program, Brno, Czech Republic; 2 Department of Neurology, St. Anne´s Teaching Hospital, Medical School of Masaryk University, Brno, Czech Republic; 3 Department of Neurosurgery, St. Anne´s Teaching Hospital, Medical School of Masaryk University, Brno, Czech Republic; 4 Institute of Scientific Instruments, Academy of Sciences of the Czech Republic, Brno, Czech Republic; Radboud University Nijmegen, NETHERLANDS

## Abstract

**Objective:**

To study the involvement of the anterior nuclei of the thalamus (ANT) as compared to the involvement of the hippocampus in the processes of encoding and recognition during visual and verbal memory tasks.

**Methods:**

We studied intracerebral recordings in patients with pharmacoresistent epilepsy who underwent deep brain stimulation (DBS) of the ANT with depth electrodes implanted bilaterally in the ANT and compared the results with epilepsy surgery candidates with depth electrodes implanted bilaterally in the hippocampus. We recorded the event-related potentials (ERPs) elicited by the visual and verbal memory encoding and recognition tasks.

**Results:**

P300-like potentials were recorded in the hippocampus by visual and verbal memory encoding and recognition tasks and in the ANT by the visual encoding and visual and verbal recognition tasks. No significant ERPs were recorded during the verbal encoding task in the ANT. In the visual and verbal recognition tasks, the P300-like potentials in the ANT preceded the P300-like potentials in the hippocampus.

**Conclusions:**

The ANT is a structure in the memory pathway that processes memory information before the hippocampus. We suggest that the ANT has a specific role in memory processes, especially memory recognition, and that memory disturbance should be considered in patients with ANT-DBS and in patients with ANT lesions.

ANT is well positioned to serve as a subcortical gate for memory processing in cortical structures.

## Introduction

Following a series of experimental and clinical studies including the large randomized controlled trial SANTE [1], ANT-DBS was introduced into the therapy of refractory epilepsy. The anterior nuclei of the thalamus (ANT) are the most well established target for DBS in the treatment of epilepsy. In epilepsy pathology the ANT is thought to function as a relay structure to amplify and synchronize epileptic activities in the circuit [2], [3]. The thalamus by its relay function of the many thalamic nuclei does appear to be a station transmitting neural signals primary to the cerebral cortex from a number of cortical and subcortical brain areas.

Interest in the cognitive functions of the ANT in human increased after the SANTE study, in which memory impairment and depression appeared as the most frequent side effects of ANT-DBS. Cognition and mood showed no group differences in objective testing, but participants in the stimulated group were more likely to report memory problems as adverse events than participants on placebo stimulation (7 vs. 1). High frequency stimulation of the ANT also disrupted the performance of memory paradigms in rats [4]. By nature, cognitive behavior such as learning and recall is highly dynamic, and therefore dynamic neuromodulatory devices are likely to alter its function. DBS (of the basal ganglia, temporal lobes, thalamus and limbic system) may also play a potentional role in positively affecting cognitive behavior such as memory formation and recall [5].

The impact of ANT-DBS on memory might be explained by the anatomical position of the ANT. The ANT belongs to the associative nuclei of the thalamus getting information from the limbic system (via the fornix and gyrus cinguli). Functionally the thalamus is connected to the limbic system by the hippocampal Papez circuit. The connection is from the hippocampus via the indusium griseum, subiculum and presubiculum to the area entorhinalis and gyrus hippocampi. Through the fornix the Papez circuit continues to the corpus mamillare and as a fasciculus mamillothalamicus to the ANT. Further, the ANT is connected with the posterior cingulate cortex and orbitofrontal cortex and via the cortex of the cingulum (area cingularis posterior) with the hippocampus directly or via the area entorhinalis. Additional circuits connect the ANT to the hypothalamus, nucleus accumbens, habenular nuclei, and the septal nuclei. The connection with the nucleus accumbens links the Papez circuit with the limbic pathway of the basal ganglia [6]. As the ANT is linked with the hippocampus, cingulate limbic structures, and the neocortex and is a key structure in the intrathalamic pathways, it is well positioned to serve the memory circuit as a relay nucleus. The extensive direct and indirect hippocampal connections support the hypothesis that hippocampus and ANT constitute a neuronal network crucial for memory [7]. The important role of the hippocampus in the memory processes is well known [8–16]. While the role of the hippocampus in the cognitive network is widely studied, less attention has been devoted to the role of the ANT in memory processing. From a clinical point of view, both the hippocampal–anterior thalamic and the perirhinal–medial dorsal thalamic systems are compromised in amnesic cases, leading to severe deficits in both recall and recognition [17]. Amnestic syndromes were observed in the pathology of the mammillary bodies, the mammillothalamic tract and the ANT [18–20]. The ANT research has focused on animal studies while it is difficult to get the data from human ANT due to the small size and position of this structure. Based on these studies it has been suggested that the ANT serve as a subcortical gate for information used in path integration processes by cortical structures [7]. We used the unique opportunity to record directly from the human ANT through the DBS electrodes as well as to explore the hippocampus by stereotactically placed depth electrodes (SEEG) to explore these two structures in memory processing.

We studied and compared local field potentials elicited by memory encoding and recognition tasks recorded in the ANT and in the hippocampus in patients with epilepsy.

We raised three questions:

1. Could local field P300-like potentials be elicited by memory tasks recorded in the ANT?
2. If so, are the ERPs in the ANT identical to or different from the ERPs recorded in the hippocampus?
3. Can the latencies of the P300-like potentials recorded in the hippocampus and in the ANT confirm that the ANT participates in preprocessing memory information?

## Materials and Methods

### Patients

Six pharmacoresistent epilepsy patients, all native Czech speakers, were included in our study. Three patients (Patients 1–3, Table 1) were implanted bilaterally with ANT-DBS (only), the other three epilepsy surgery candidates had depth electrodes (SEEG) implanted bilaterally in the hippocampi (Patients 4–6, Table 1). No patient had electrodes implanted in the hippocampus and ANT simultaneously, as there was no medical reason for that. Therefore the direct comparision could not be made for the same individuals but only interindividually.

**Table 1:** The main characteristics of patients with ANT and hippocampal electrodes.

Patient	1	2	3	4	5	6
**Age**	47	29	35	29	29	54
**Sex**	F*	M**	M	M	F	F
**Laterality**	right-handed	right-handed	left-handed	right-handed	right-handed	right-handed
**Type of epilepsy**	Multifocal non lesional	Temporal lobe epilepsy (no further specification possible)	Epilepsy of the left frontal lobe	Extratemporal epilepsy probably of the occipital lobe (left)	Right temporal lobe epilepsy	Left temporal lobeepilepsy
**MR of the brain results**	negative	negative	Laminar heterotopia of the fronto-parietal region bilateral	negative	DNET*** of the right temporal lobe (amygdala)	negative
**Memory testing (before the surgery)**	average MQ****: 95	Visual memory: average, verbal: slightly below average MQ: 95	average MQ: 96	average MQ: 97	average MQ: 99	average MQ: 105
**IQ** *****	IQ: 108	IQ: 97	IQ: 88	IQ: 108	IQ: 86	IQ: 103
**Position of the electrodes**	ANT******	ANT	ANT	Hippocampus	Hippocampus	Hippocampus
**Medication**	Lamotrigine, Clonazepam	Topiramate	Lamotrigine, Levetiracetam, Eslicarbazepin, Clonazepam	Levetiracetam, Pregabalin	Lamotrigine, Levetiracetam	Levetiracetam

*F:female

**M:male

***DNET:Dysembryoplastic neuroepithelial tumor

****MQ:memory quotient

*****IQ: intelligence quotient

******ANT: anterior nuclei of the thalamus.

All patients underwent complex presurgical evaluation including advanced diagnostic methods (MRI, PET: Positron Emission Tomography, SPECT: Single-Photon Emission Computed Tomography, and SISCOM: subtraction of ictal and interictal SPECT coregistered to MRI) and complex neuropsychological testing (Wechsler Adult Intelligence Scale–III, event. Wechsler Adult Intelligence Scale Revised (short form with subtests: picture completion, arithmetic, similarities, digit symbol—coding, digit span), Rey-Osterrieth Complex Figure Test, Wechsler Memory Scale—III (Word List subtest), Stroop Test, Verbal Fluency Test, Zung Depression Scale, and Hamilton Anxiety Scale). Neuropsychological examinations conducted prior to the implantation showed slight impairments in various domains with no signs of dementia or psychiatric or behavioral disorders.

We compared the ANT-DBS group with the group of pharmacoresistent epilepsy patients with deep brain electrodes (SEEG) implanted bilaterally in the hippocampi. These patients underwent invasive video EEG exploration before the epilepsy surgery which followed. The ANT-DBS was recommended by the Epilepsy Surgery Commission of the Brno Epilepsy Center and it was considered as the last available option for patients in whom all other therapies failed. None of the patients had responded to long-term vagus nerve stimulation (VNS). The VNS devices were explanted before the DBS implantation. The study and the recordings were approved by the local ethics committee (St. Anne´s Ethics Committee) and the patients gave their written informed consent. The patients were simultaneously treated with the current dosage of their antiepileptic medication (Table 1).

### Surgical methods and procedure

#### DBS procedure

All three patients with ANT-DBS underwent implantation using the ceramic stereotactic Leibinger open frame with the Praezis Plus software and the Talairach and Schaltenbrand Bailey atlases.

**Fig 1:**
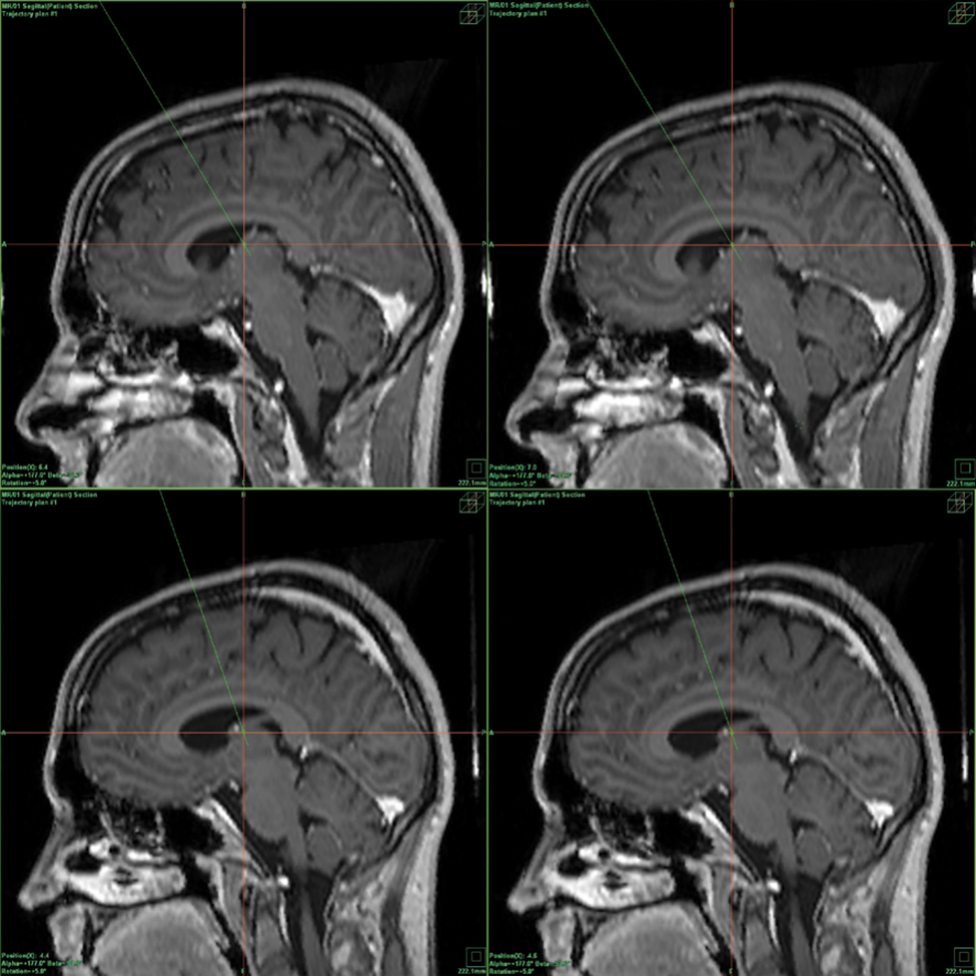
Example of the actual positions of the all ANT contacts in patient 2 (right-left orientation of the sagittal scans, fusion of the MR scans and CT correlation). Upper picture: position of the right ANT contacts: the R3 contact (left picture), and position of the R4 contact (right picture). Lower picture: position of the left ANT contacts: the L3 contact (left picture) and position of the L4 contact (right picture).

The initial coordinates for the ANT as related to the anterior commissure- posterior commissure (AC-PC) line centre were 0–2mm anterior to the midpoint, 5.5mm laterally, and 10–12mm above the AC-PC line. The entry point for the electrode was planned at the proximity of the coronal suture. The final target was modified according to local anatomy, and particular attention was paid to the safe distance of the trajectory from the thalamostriate vein and choroid plexus. All four electrode contacts were planned to be inside thalamic structures. The stimulation leads (Medtronic, Inc.) were implanted bilaterally into the targeted structure using a stereotactic magnetic resonance imaging (MRI)-guided technique and local anesthesia. Intraoperative microrecordings to guide lead placement were employed. Intraoperative stimulation was used to test for possible adverse effects. Once the final target coordinates were defined, a permanent quadripolar DBS electrode (model 3389, with 1.5mm contact length and 0.5mm intercontact distance) was implanted. The electrode position was verified by the intraoperative skiascopy control using the C-arm with postoperative confirmation of electrode position (lateral and anteroposterior radiographs under stereotactic condition and postoperative CT with stereotactic frame and markers). After surgery completion, CT scans under stereotactic conditions covering the entire length of the implanted electrodes were added. The series of images were reimported to the planning workstation and subsequently the coordinates were correlated with the actual positions of the implanted electrodes (Fig 1). Any potential deviation of the final electrode position is readily observed after this correlation without being burdened by the material artifacts. The positions of the electrodes and their contacts in the brain were also later verified using post-placement MRI with electrodes in situ. DBS electrodes were first connected to extracranial extensions (the connection being subcutaneous) enabling recordings from the DBS contacts. After 3–4 days of video-EEG monitoring, the extracranial extensions were removed and the DBS electrodes were connected to the implanted battery.

#### SEEG procedure

The patients with hippocampal target underwent an exploration of the epileptic focus (seizure onset zone) via stereotactically implanted depth electrodes. The patients were implanted with 6, 9 and 14 orthogonal electrodes respectively using the Talairach and Bancaud methodology [21] to explore all possible seizure onset zones. Five-to fifteen- contact platinum semiflexible Alcis (Besançon, France) electrodes, each with a diameter of 0.8mm and a contact length of 2mm, and with intercontact intervals of 1.5mm, were used. The exact positions of the electrodes were verified using post-placement MRI with electrodes in situ. The electrodes were implanted bilaterally in the hippocampi (as well as in other target structures that were not included in our study). The study was approved by the local ethics committee. All three patients were fully informed about the character of the study and gave their informed consent. The patient characteristics are shown in the Table 1. The patients later underwent focal cortectomy of the seizure onset zone based on the SEEG findings combined with all non-invasive methods (MRI, PET: Positron Emission Tomography, SPECT: Single-Photon Emission Computed Tomography, SISCOM: subtraction of ictal and interictal SPECT coregistered to MRI), neuropsychological testing, and clinical findings from standard neurological examinations, used in the preoperative phase in order to localize the seizure onset zone. In MRI, as our standard protocol in epilepsy patients, we used T1 and T2 weighted imaging, T2 FLAIR (fluid attenuation inversion recovery) and inverse T2, T1 and T2 coronal and coronal FLAIR scans, DWI (diffusion weighted imaging), T2 FRFSE (with fat suppression), T2 GRE (gradient recalled echo), T1 FSPGR (ultrafast spoiled gradient echo), T2 and PD (proton density) FSE (fast spin echo) on 1.5 Tesla MR.

### Recordings

The episodic memory testing was conducted during the encoding and recognition phases in visual and verbal modalities. Patients were tested in two consecutive days. Visual testing was performed on the first day and verbal (auditory) testing on the second day. The elaborations of the tests for this study were based on protocols used in an intracranial recording study by Jones-Gotman et al. [22] and on protocols used in an fMRI study by Rabin et al. [23]. The first results obtained in our laboratory were published by Štillová et al. [24].

For the visual task, the patients were seated in front of a computer monitor at a 1.5m distance from the screen. In the encoding phase, 30 emotionally neutral photos were presented for 2 seconds each. The photos showed static scenes, (nature, towns, etc.). During the interstimulus interval, a black screen appeared for 4 seconds. The patients were instructed to remember the presented pictures.

After the encoding phase, a 15-minute break followed, during which the patients watched a well-known fairy tale. They were asked not to remember the story, only to relax. The recognition phase followed: 60 photos were presented, with 30 pictures that had been seen in the encoding phase and 30 new pictures. The pictures appeared on the monitor in random order. The patients held a two-button device in their right hand. They were asked to push the right button when a picture was recognized as having been presented before or the left button when the picture was considered to be a new one.

The verbal memory task was tested the next day. The patients were seated in front of a computer at a 1.5m distance and listened to words through the speakers. In the encoding phase, 30 words were presented with a silent interstimulus interval of 4 seconds. Both abstract and concrete words were intermingled. The words were two or three syllables long, emotionally neutral, and commonly used in the Czech language. The patients were instructed to remember the presented words.

A 15-minute break followed, during which the patients watched a well known fairy tale. The recognition phase followed: 60 words were presented, with 30 words that had been heard in the encoding phase and 30 new words. The old and new words were presented in random order. Using the same device as for the visual task, the patients were asked to push the right button when a word was recognized as having been presented before or the left button when the word was considered to be a new one [24].

The recordings from hippocampal electrodes were performed with the TruScan system (Deymed Diagnostic, Alien Technic) 128-channel EEG machine. The sampling frequency was 1024 Hz with standard anti-aliasing filters.

The recordings from ANT electrodes were performed with the M&I EEG system. The scalp EEG was recorded simultaneously. The recordings were made in a monopolar montage with connected earlobes used as a reference. The signal was filtered in the range from 0.2 to 200 Hz in the time base 2 seconds before the stimulus and 5 seconds after.

### Data evaluation and analysis

**Fig 2:**
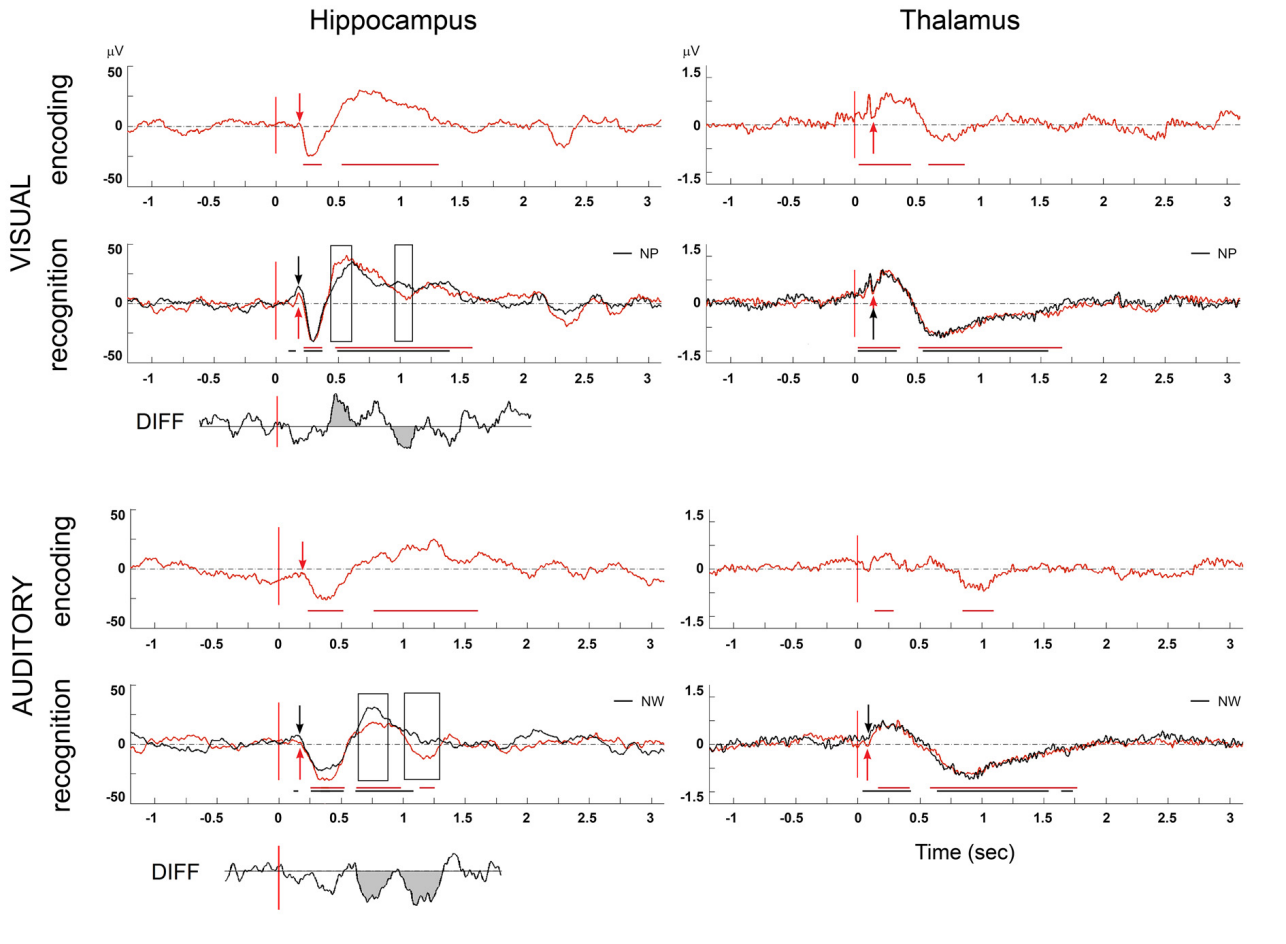
P300-like potentials in the hippocampus and the thalamus (the mean from all hippocampal and thalamic contacts from all patients in bipolar montage during the encoding and the recognition task). The black curve indicates a new stimulus (new picture/word: NP/NW); red indicates a repeated stimulus during the recognition phase (old picture/word: OP, OW). The arrow shows the beginning of the P300-like potential (red for the repeated stimulus, black for the new one.) Axis *x* is time (in s); axis *y* is amplitude in μV. The statistical significance to baseline is highlighted by black and red horizontal lines. The significant difference between tasks in the hippocampus is highlighted by a black rectangle. Simultaneously the difference between tasks is drawn—DIFF.

The data analysis, segmentation, and evaluation were made by using the ScopeWin and MATLAB software systems. The data were segmented according to the stimulus onset (time 0 in figures), and all the trials were visually controlled. Trials with epileptiform activity or other technical problems were excluded. The trial length was 7s: 2s before and 5s after the onset of the stimuli. The baseline interval was determined 600–100 ms before the stimulus occurred. The mean values from the baseline intervals were subtracted within each trial. After trend elimination in each trial, data were filtered with a 0.2–40 Hz band pass and artifact-free trials were averaged. The statistical significance of the differences between the mean during the baseline interval and the mean computed from the 150ms length sliding window after stimuli is expressed by the probability p value. The same sliding window was used when inter-task differences were computed. We used the non parametric Wilcoxon Rank Sum (Signed Rank) test for paired samples in each trial. The amplitude changes after stimulus were considered significant when the probability value p was lower than 0.05. The statistical significance to baseline is highlighted by a horizontal bar (black and red) in the figures (Fig 2). The differences between stimuli were analysed with an unpaired t-test in a time interval 0–1.5s. The level of significance was designated at a p value less than 0.01 two-tailed test was used. The significance between stimuli is shown in figures as delimited by horizontal bars. The local field potential was verified by a bipolar montage for each electrode in every task. The bipolar montage evaluation was used to exclude the volume conduction from other structures, namely from the cortex or transsynaptic propagation along cortical-subcortical pathways [25–26] and confirm the local origin of the potentials. Contacts in the thalamus were placed very close together. Any EEG signal from the common reference was eliminated by a bipolar montage. Even minor bipolar montage activity displays the origins of detected activity in the ANT. The main ERP components in the 200–450 ms following stimuli were identified by visual inspection and quantified by latency and amplitude measures (P300-like potential). The peak latencies were measured from stimulus onset (point 0). The intracerebral potentials occurred with both positive and negative polarities. This was due to variances in the positions of the electrode contact and of the dipole generator. Absolute amplitudes were measured from the baseline. The distance from the electrode to the generator heavily influences the amplitude of intracerebral recorded potentials, and thus the differences of amplitude can only be compared intraindividually.

The bootstrap methodology [27] with 500 repetitions was used to assess the mean latency and corresponding distribution of P300-like potentials for different tasks. The statistical significance of differences among latencies was tested by the Wilcoxon rank sum test and the Bonferroni correction was used (Table 2).

**Table 2:** P300-like potentials: latency of P300-like potentials recorded from all contacts from the hippocampus versus all contacts from the ANT, mean and standard deviation.

Structure	Hippocampus Latency (ms)	Diff	ANT Latency (ms)
**Visual encoding**	285±20	NS	240±60
**Visual recognition**	NP: 322±4	***	NP: 278±14
	OP: 321±4	***	OP: 273±7
**Diff NP vs. OP**	NS		*
**Verbal encoding**	376±34	***	No significant potential
**Verbal recognition**	NW: 376±25	***	NW: 274±32
	OW: 378±17	***	OW: 253±31
**Diff NW vs. OW**	NS		**

OP/ OW: old picture/ word, NP/NW: new picture/word.

Statistical significance of differences between Hippocampus and ANT is in column Diff. Statistical significance of differences between the old and new pictures/words is in line of Diff NP vs. OP or Diff NW vs. OW. Significance of differences

P<0.001 ***

P<0.01 **

P<0.05 *

NS: non-significant.

## Results

We analyzed recordings from the contacts placed in the thalamus, focusing on the anterior nuclei. We compared the recorded data from the thalamus with the data from hippocampal electrodes. The accuracy rate of correct recognition by the patients (characteristics of the patients shown in Table 1), patient 1: 84%, patient 2: 79%, patient 3: 83%, patient 4: 84%, patient 5: 90%, and patient 6: 84.5% of the correct answers.

### ERP analyses

**Fig 3:**
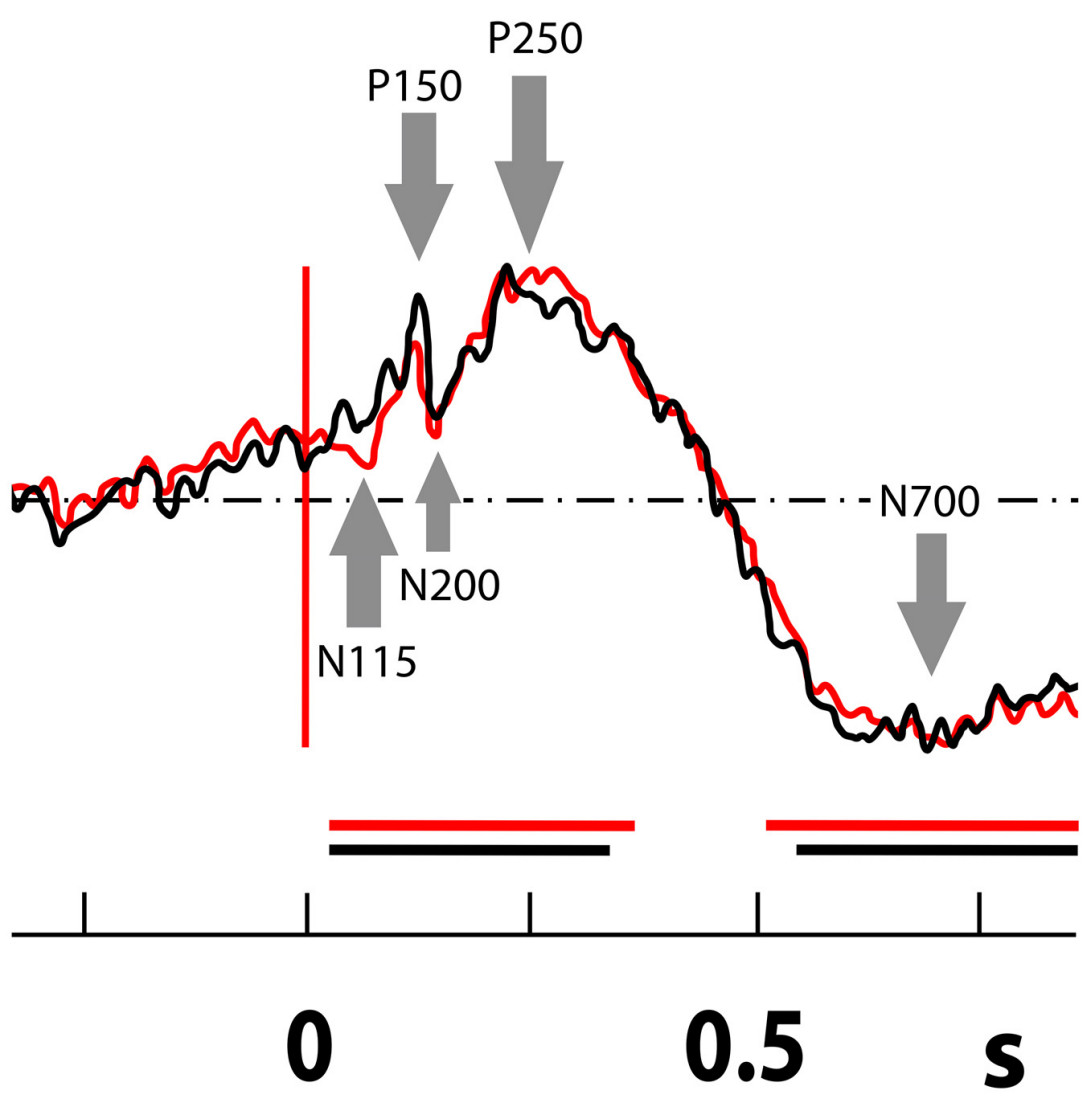
Visual recognition task in the ANT: N 115- P 150- N 200- P 250 and N700 complex (the mean from all thalamic contacts from all patients in bipolar montage during the recognition task). Arrows mark fast and slow ERP components N115, P150, N200, P250, and N700. The black curve indicates a new stimulus (new picture); red indicates a repeated stimulus during the recognition phase (old picture). On axis *x* is time (in s). The statistical significance to baseline is highlighted by black and red horizontal lines.

ERPs elicited by the visual encoding task and the visual and verbal recognition tasks were recorded in the ANT. In the visual encoding and recognition tasks an N 115- P 150- N 200- P 250 and N700 complex was detected (Fig 3). The first two waves (N115-and P150) probably represent a visual evoked potential (VEP): such a potential was not recorded in the ERPs detected during the verbal tasks in the ANT. The verbal encoding task elicited only a small late potential peaking at about 1sec; no significant ERP was detected in the period typical for cognitive response.

In the hippocampus the ERPs were elicited by all tasks. An N200- P300- P750 like complex was detected in the hippocampus in both the visual and verbal recognition stimuli tasks. The latencies in the verbal stimuli tasks were longer than in the visual tasks. The early visual potentials with latency about 150 ms that were observed in the ANT were not recorded in the hippocampus. In both recognition tasks (visual and verbal) the latency of the P300-like potentials in the ANT was shorter than in the hippocampus, using the bootstrap for statistical significance (Fig 2, Table 2).

The latency of P300-like potential detected in the ANT was significantly shorter (P<0.001) than the latency of P300-like potential in the hippocampus in all cases except visual encoding, where the latency was shorter, but not with statistical significance. Comparing the difference in P300-like potential latencies between the old (repeated) stimulus and the new stimulus during the recognition phase, the ERP latency in the ANT corresponding to the new stimulus was longer (P<0.05) than the latency corresponding to the old stimulus. There were no differences in the hippocampus between old and new stimuli.

Although the seizure onset zones in all patients were localized in different areas, the patients had different structural MR findings and were on a different antiepileptic medications, we believe that the observed ERPs were not fundamentally affected by any pathological processes or by the type of the medication taken at the time of our study.

## Discussion

In recent years, there has been extensive scientific concentration on the cortical structures as primary structures for cognition and behavior, and mainly as the primary structures responsible for regulation. Recent studies have made it increasingly obvious that it is also important to focus on subcortical brain structures including the thalamus. Our results confirm the role of the hippocampus in memory encoding and recognition processes and reveal a selective participation of the ANT in these tasks. The activation of the ANT appears to be mode and task dependent. The ANT participates in the pre-processing of memory tasks, preceding the hippocampal activity. Traditionally, the cognitive networks have been considered to be the cortical network. Far less attention has been paid to the cognitive role of the subcortical structures. Parvizi [28] called this situation “corticocentric myopia”. The ANT is not only connected with cognition, but is also involved in a complex visuomotor task, as was reported recently in humans [29].

The importance of the anterior thalamus in human memory is becoming indubitable [30–32]. Damage to the anterior and medial parts of the thalamus, including the ANT, mediodorsal, midline and intralaminar nuclei can contribute to amnesia, although the nature of the memory deficit may vary. This was verified in animal models [4], [17], [31], [33–35] and in clinical practice in a published study of a group of patients with amnestic syndrome caused by thalamic infarction [20], [36].

The ANT was suggested as a critical nodal point in an extended hippocampal system in spatial and non-spatial memory [17], [7], [37], [38]. Several studies focused on the hippocampal-ANT cooperation and on the direct and indirect influence of the ANT on the hippocampus. The involvement of the ANT and the hippocampal-anterior thalamic interconnections in human episodic memory and rodent event memory is known. The hippocampal-ANT axis is considered to be important for memory recall [31]. The role of the ANT in episodic memory in terms of learning of visual discrimination (object in place) was found in monkeys by Parker and Gaffan [33] and the selective activation of the ANT during the retrieval phase of memory recognition was observed in an fMRI study in humans [39]. Our results suggest a specific role of the ANT in memory recognition processes and as a recognition “pre-processor” before the information gets further to the hippocampus.

Our previous SEEG recordings with identical tasks displayed similar results, i.e., activity linked with recognition in both modalities and absence of activation with the verbal encoding in the posterior medial cortex, namely the posterior cingulate cortex (PCC) and precuneus [24]. In animal studies the ANT contributes to modulating plasticity in the PCC [40]. PCC activity decreases following thalamic lesion in humans [41], [42]. The PCC was also more active during retrieval in a study by Pergoli [39] which supports the interpretation that an ANT-PCC network is critical for the retrieval phase of recognition accompanied by recall. The posterior cingulate gyrus has a bidirectional connectivity via the cingulum with the hippocampus [43], and the ANT is connected via the PCC with the hippocampus. Simple visual, but not verbal evoked potentials were recorded in the ANT. No such potentials were recorded in the hippocampus. It seems that the ANT may participate in the visual system. Further studies are needed in order to understand the nature of this involvement.

## Conclusion

The ANT is a subcortical part of the cortico-subcortical memory network, possibly with particular functional relation to the posterior medial cortex. Our results confirm the importance of the anterior thalamus in cognitive functions, its specific role in memory recognition, and a selective, modality-dependent role in memory encoding. There is a strong implication that the functions of the ANT in memory processing are not only driven by the hippocampus but that actions in the opposite direction may by equally crucial [7]. ANT is well positioned to serve as a subcortical gate for further memory processing in cortical structures.
